# Evaluating Vulnerability and Resilience of Mediterranean Sharks, Rays and Chimaeras Through Biological and Fishery Traits

**DOI:** 10.3390/biology15211931

**Published:** 2026-10-24

**Authors:** Cemal Turan, Alen Soldo, Ali Uyan

**Affiliations:** 1 Molecular Ecology, Genomics and Computational Biology Laboratory, Molecular Biology and Genetics Department, Faculty of Engineering and Natural Sciences, Atlas University, 34403 Istanbul, Türkiye; 2 Faculty of Marine Science, University of Split, 21000 Split, Croatia; 3 Fisheries Faculty, Mersin University, 33160 Mersin, Türkiye

**Keywords:** elasmobranchs, index of resilience and vulnerability, life-history traits, Mediterranean Sea, risk assessment

## Abstract

Mediterranean sharks, rays, and chimaeras face substantial conservation pressures, yet comparative regional assessments integrating life-history, ecological, and fisheries-related traits remain limited. We compiled and standardized 1634 species-specific data points for 80 species and applied a multi-criteria framework to estimate resilience (R) and vulnerability (V), followed by an Index of Resilience and Vulnerability (IRV) assessment. Based on the predefined trait framework, 46 shark, 32 ray, and 2 chimaera species were assessed. Mean R and V scores were 1.80 and 2.27 for sharks, 1.85 and 2.38 for rays, and 1.64 and 2.10 for chimaeras, respectively. Mean IRV was highest in sharks (0.17), followed by rays (0.16) and chimaeras (0.16). Across all species, R scores ranged from 1.29 to 2.57, V scores from 1.60 to 3.00, and IRV scores from 0.05 to 0.37. No species occurred in the comparatively high-resilience, low-vulnerability region of the R–V space. Several shark species exhibited particularly low IRV scores, while *Dipturus nidarosiensis* and *D. batis* showed the highest vulnerability among rays. IRV increased with R and decreased with V, as expected from the mathematical construction of the index. Comparisons with FishBase revealed broadly similar vulnerability patterns, whereas the IRV scores provided a complementary continuous perspective on species-level resilience and vulnerability. The IRV framework provides a comparative trait-based approach for regional prioritization of Mediterranean cartilaginous fishes.

## Simple Summary

Sharks, rays, and chimaeras are important members of marine ecosystems, but many species are increasingly affected by fishing pressure, slow population growth, and other human-related threats. In this study, we brought together information on the biology, ecology, distribution, and fisheries of 80 species occurring in the Mediterranean Sea. We used these data to compare how well species may recover from population declines and how strongly they may be affected by environmental and fishing pressures. Our results show that many Mediterranean cartilaginous fish species combine a low capacity for recovery with high vulnerability, and none of the assessed species showed a clearly favourable combination of high recovery potential and low vulnerability. Sharks generally showed the greatest overall concern in the combined assessment, although several ray species were also highly vulnerable. The results showed broadly similar vulnerability patterns to existing global assessments but also highlighted species that may deserve closer regional attention. This study provides a practical way to compare species using available biological and fisheries information and can help scientists and managers identify species that may require greater monitoring, research, and conservation attention in the Mediterranean.

## 1. Introduction

Cartilaginous fishes (Class Chondrichthyes), comprising sharks, rays, and chimaeras, represent an ancient and evolutionarily distinct group of vertebrates that play a critical role in maintaining the structure and function of marine ecosystems [1,2]. As apex and mesopredators, they exert top-down control on prey populations, contributing to the stability of marine food webs and influencing community dynamics through trophic interactions [3,4,5,6,7]. Despite their ecological importance, cartilaginous fishes are among the most threatened marine vertebrates globally, with an increasing number of species facing significant population declines due to fishing-related pressures, including overfishing and bycatch [8,9,10,11,12].

The Mediterranean Sea, although representing less than 1% of the world’s ocean surface, harbours a high diversity of marine species, including over 80 species of cartilaginous fishes [1,2,13]. However, this region is also characterized by some of the highest levels of marine resource exploitation globally [1]. Historical and ongoing fishing pressures, coupled with the basin’s semi-enclosed nature and high coastal population density, have placed Mediterranean elasmobranchs under severe and sustained threat [2,14,15,16,17]. According to the International Union for Conservation of Nature (IUCN), 39 out of 73 assessed species are considered regionally threatened (Critically Endangered, Endangered, or Vulnerable), while 13 species are considered Data Deficient, with many exhibiting evidence of local extirpations or severe population declines [1].

A key factor underlying the vulnerability of cartilaginous fishes is their suite of conservative life-history traits, which include slow growth rates (K), late sexual maturity, long gestation periods, and low fecundity [1,2]. These characteristics result in inherently low population productivity and a limited capacity for rapid recovery following exploitation [18]. Thus, species with such biological constraints are particularly susceptible to overfishing and environmental perturbations, and require targeted, trait-informed management approaches to ensure their conservation.

The use of species’ bio-ecological and distributional characteristics has been found to be very important in determining the sensitivity of species to climate change and human activities and their adaptive capacity [19,20,21]. In particular, the loss of species with unique functional traits can have more serious consequences on ecosystem function than the loss of species with more common traits. Within the Index of Resilience and Vulnerability (IRV) framework, resilience denotes the capacity for relative biological recovery, whereas vulnerability represents the composite V dimension. These analytical terms are distinct from the formal IUCN Red List categories. Species may show different levels of resilience and vulnerability depending on their ecological and life-history characteristics and their responses to pressures such as climate change and fishing.

While species-specific risk assessments and conservation prioritization efforts have increased in recent years, there remains a pressing need for integrative, trait-based frameworks that explicitly incorporate both biological and fisheries-related data [21,22,23]. Biological traits reflect a species’ natural ability to recover but do not account for its exposure to fishing. To address this, fisheries-related factors were added to capture other aspects of vulnerability tied to how species interact with fisheries [19,20]. Therefore, a comparative assessment is needed to identify Mediterranean cartilaginous fishes characterized by high vulnerability and low resilience within the IRV framework.

Several trait-based approaches have been developed to evaluate species-level vulnerability in relation to fisheries. Among these, Productivity–Susceptibility Analysis (PSA) combines biological productivity with susceptibility to a particular fishery or fishing activity [24,25]. The Index of Resilience and Vulnerability (IRV), in contrast, separately evaluates biological resilience and vulnerability derived from fisheries-related, distributional, reproductive, and population-status attributes before integrating these dimensions for comparative species-level prioritization [19]. Although PSA and IRV may use some overlapping biological and fisheries-related traits, they differ in their analytical structure and intended application. Whereas PSA is generally applied to assess risk in relation to a defined fishery, IRV characterizes relative differences in resilience and vulnerability among species within a regional assemblage.

Therefore, this study aims to (i) compile and standardize Mediterranean-specific data on the life-history, ecological, and fisheries-related traits of sharks, rays, and chimaeras; (ii) evaluate species resilience (R) and vulnerability (V) using a multi-criteria comparative framework; and (iii) use the IRV approach to identify species with low biological resilience and high vulnerability. The findings of this study are expected to support evidence-based policy development, enhance the effectiveness of regional fisheries management, and contribute to the long-term conservation of Mediterranean cartilaginous fishes in the face of intensifying anthropogenic pressures and environmental change.

## 2. Materials and Methods

### 2.1. Documents Collection

Species-specific biological, ecological, distributional, and fisheries-related parameters were compiled through a structured literature survey and database screening conducted up to August 2025. Data were obtained from peer-reviewed publications identified primarily through Web of Science and Google Scholar, as well as from FishBase [26], the International Union for Conservation of Nature (IUCN) Red List [27], Food and Agriculture Organization (FAO) reports [28], and region-specific fisheries literature and datasets. FishBase records were not used directly without verification. Each extracted value was cross-checked against the cited primary source when available and evaluated for taxonomic accuracy, relevance to the Mediterranean Sea, unit consistency, duplication, and biological plausibility. Only records satisfying these criteria were retained in the final dataset.

The literature search was conducted using combinations of terms including “shark”, “ray”, “chimaera”, “Mediterranean”, “life history”, “growth”, “fecundity”, “trophic level”, and “fishery interaction”, together with species-specific scientific names where appropriate. Searches focused on records reporting biological, ecological, distributional, reproductive, or fisheries-related information relevant to Mediterranean species or documented occurrences. Studies were included when they provided species-specific information that could be assigned to one or more of the predefined resilience or vulnerability attributes. Records were excluded when species identification was uncertain, the reported parameter could not be reliably assigned to a species, the information was not relevant to the Mediterranean assessment, or the value could not be standardized or verified. When several publications reported the same underlying observation, duplicate records were identified and treated as a single source observation where appropriate.

For inclusion in the Mediterranean species assemblage, a species was required to have at least one documented occurrence or historical record from the Mediterranean Sea supported by a published source or a recognized biodiversity or fisheries database. This criterion was based on documented regional occurrence and did not require evidence of an established, resident, or self-sustaining population. Accordingly, species represented by rare, occasional, sporadic, or otherwise infrequent Mediterranean records were not excluded a priori, provided that their regional occurrence was documented and sufficient biological or fisheries-related information could be verified for the predefined trait-based assessment. Likewise, non-indigenous or Lessepsian species were eligible when their occurrence in the Mediterranean was documented. Conversely, species for which no reliable Mediterranean occurrence record could be identified were excluded. Importantly, inclusion in the assessment should not be interpreted as evidence of permanent residency, population establishment, demographic stability, or long-term persistence within the Mediterranean Sea.

For parameters reported from multiple Mediterranean locations or populations, the geographic origin and reported value were retained during data compilation. When multiple verified estimates were available for the same species and parameter, values were converted to a common unit and combined to obtain a representative species-level estimate, as described in Section 2.2. Thus, the final analytical dataset represents standardized species-level values rather than an unprocessed compilation of independent observations. Geographic occurrence status was used as an inclusion criterion for the regional assemblage but was not itself incorporated as an additional numerical component of the resilience or vulnerability scores.

In total, 413 studies were reviewed and screened according to the above criteria, resulting in 1634 verified parameter entries for 80 Mediterranean cartilaginous fish species. The search and screening process was designed to maximize consistency and biological relevance while retaining the widest possible coverage of available Mediterranean-specific information.

### 2.2. Preparation of Data

The compiled dataset was structured as a species–trait matrix with rows representing species and columns corresponding to the biological and fishery-related parameters defined within the IRV framework [19,24,25]. During data extraction, individual estimates were retained together with their source, geographic origin, sampling period, sample size, and sex-specific information when these were reported in the original study. When multiple verified Mediterranean estimates were available for a given parameter, values were first checked for comparability and standardized to a common unit before being averaged to obtain a representative species-level estimate [29]. Estimates reported for different populations or sampling locations were therefore aggregated into a single comparative value for each species.

Where sex-specific estimates were reported, these were retained during data extraction and considered as separate reported estimates before species-level aggregation, rather than assuming that one sex was representative of the species. Similarly, estimates obtained from different sampling periods or geographically separated Mediterranean populations were retained as independent reported observations and subsequently combined only when they represented the same predefined biological trait and could be reliably standardized. Growth estimates obtained using different model formulations or parameterization procedures were considered according to the biological parameter reported and were included only when the resulting estimate could be reliably assigned to the corresponding trait used in the IRV framework [19,24,25,29]. In total, the dataset comprised 1634 parameter entries across 80 species, representing a standardized comparative framework integrating heterogeneous biological and fisheries-related information. All calculations were performed using RStudio v4.3.2 [30].

The arithmetic mean was used as the aggregation rule when multiple comparable and verified estimates were available for the same species and trait. Estimates were not weighted by sample size, geographic area, population size, or study period because consistent information required for such weighting was not available across the full Mediterranean dataset. The use of an unweighted mean was therefore intended to provide a transparent and reproducible species-level summary for the comparative IRV framework, rather than to estimate a population-specific demographic parameter [19,29]. The resulting species-level values should consequently be interpreted as representative comparative trait estimates rather than as estimates of a particular Mediterranean population.

The same standardized scoring framework was applied consistently to all assessed sharks, rays, and chimaeras, following the comparative trait-based approach described in previous studies [19,26,31]. Each species was evaluated using the predefined seven resilience (R) traits and five vulnerability (V) traits. For each trait, the corresponding species-specific value was converted into an ordinal score of 1–3 according to the predefined thresholds presented in Table 1. These thresholds were established separately for sharks, rays, and chimaeras to account for differences in the biological ranges of the three taxonomic groups [19,26].

The composite resilience score (R) was calculated from the seven resilience-trait scores, whereas the composite vulnerability score (V) was calculated from the five vulnerability-trait scores. Thus, the same predefined trait structure and scoring procedure were applied across all species included in the analysis. No numerical values were imputed to replace missing biological observations. Species were retained for the IRV assessment when the available verified information permitted assignment of the required trait scores for both R and V components [19,24,25]. The resulting scores therefore represent standardized relative positions within the assessed Mediterranean assemblage rather than absolute demographic or extinction-risk estimates [19,31,32].

**Table 1:** Ranking criteria and explanation of different resilience and vulnerability attributes used for sharks, rays and chimaeras in IRV.

R Score		Criteria				Explanation
		Rank	Shark	Ray	Chimaera	
1	Growth coefficient (*K*)	1	≤0.1	≤0.1	≤0.1	Species exhibiting high *K* values are more resilient.
		2	≤0.2	≤0.2	≤0.2	
		3	>0.2	>0.2	>0.2	
2	Asymptotic length (*L_∞_*)	3	≤400	≤70	≤50	Small-sized species showing lower *L_∞_* values are more resilient.
		2	≤800	≤100	≤75	
		1	>800	>100	>75	
3	Length at maturity (*L_m_*/*L_∞_*)	3	≤0.1	≤0.1	≤0.1	This ratio indicates the reproductive burden of the species. Earlier maturation (lower *L_m_*/*L_∞_* ratio) is associated with higher resilience.
		2	≤0.2	≤0.2	≤0.2	
		1	>0.2	>0.2	>0.2	
4	Number of spawning months	1	≤4	≤4	≤4	Species with longer spawning periods are assessed more resilient.
		2	5–8	5–8	5–8	
		3	9–12	9–12	9–12	
5	Gestation period	3	≤4	≤4	≤4	Shorter gestation enhances resilience, boosts reproductive rates, and enables faster recovery from disturbances.
		2	≤8	≤8	≤8	
		1	>8	>8	>8	
6	Fecundity (Fc)	1	≤5	≤5	≤5	Species with high fecundity are more resilient.
		2	≤10	≤10	≤10	
		3	>10	>10	>10	
7	Mean trophic level (MTL)	3	≤2.5	≤2.5	≤2.5	Lower trophic level fishes are more resilient.
		2	<3.5	<3.5	<3.5	
		1	≥3.5	≥3.5	≥3.5	
V Score		Rank	Shark	Ray	Chimaera	Explanation
8	Species geographic Distribution (Dist)	1	=3	=3	=3	Species with broader ranges are less vulnerable. Here, geographic distribution was estimated from Mediterranean occurrence records.
		2	=2	=2	=2	
		3	=1	=1	=1	
9	Length at recruitment Lc/L∞ ratio	3	≤0.3	≤0.3	≤0.3	Represents the relative size at which individuals become susceptible to fishing gear.
		2	≤0.5	≤0.5	≤0.5	
		1	>0.5	>0.5	>0.5	
10	Gear susceptibility (G)	3	=3	=3	=3	Gear susceptibility was assessed as species-specific interaction with fishing gears, based on general selectivity and catchability patterns, following Bjordal [33].
		2	=2	=2	=2	
		1	=1	=1	=1	
11	Age at first sexual maturity	1	≤5	≤4	≤4	This ratio indicates the first reproductive onset of the species. Species maturing later are considered more vulnerable.
		2	≤15	≤8	≤8	
		3	>15	>8	>8	
12	Population size	1	=1	=1	=1	Population size used as an indicator due to limited abundance data for Mediterranean cartilaginous fishes. Using data from the IUCN Red List [27], including species distribution patterns and overall status assessments, species were classified into three population categories: abundant (1), medium-sized (2), and small (3).
		2	=2	=2	=2	
		3	>2	>2	>2	

### 2.3. Resilience and Vulnerability Calculations

Resilience and vulnerability were assessed as distinct but interacting dimensions within the IRV framework described by Mohamed et al. [19]. Resilience was operationally defined as the relative biological recovery capacity of a species, as inferred from selected life-history and ecological traits [19,31,32]. Vulnerability was defined as a composite dimension incorporating fisheries-related, distributional, reproductive, and population-status attributes [19,24,25].

The present assessment was designed as a regional species-level comparison rather than a Productivity–Susceptibility Analysis (PSA) of a defined fishery. Accordingly, the R and V scores should not be interpreted as conventional PSA productivity and susceptibility scores. Traits were scaled separately for sharks, rays, and chimaeras as 1 (low), 2 (medium), or 3 (high), following previous comparative trait-based assessments [19,26,31]. The scoring thresholds were established to provide a standardized ordinal classification of resilience and vulnerability among Mediterranean sharks, rays, and chimaeras. The direction of scoring for each trait was determined according to its expected biological relationship with resilience or vulnerability, following the conceptual framework of Mohamed et al. [19] and previous trait-based assessments [26,31]. Thresholds were adapted to the biological characteristics and observed trait ranges of Mediterranean Chondrichthyes rather than being transferred uncritically from the tropical fish dataset of Mohamed et al. [19]. For traits with biologically constrained ranges, the biologically meaningful range was considered when defining the ordinal categories. In particular, the number of spawning months (NSM) was treated within the biologically possible annual range of 1–12 months, with longer spawning duration representing greater resilience. Accordingly, NSM values were classified as ≤4 months (rank 1), >4–8 months (rank 2), and >8–12 months (rank 3), with year-round spawning (12 months) representing the highest resilience category [19]. The resulting thresholds and their biological interpretation are presented in Table 1.

Importantly, thresholds for chimaeras were not derived by subdividing the observations of the two assessed species into three statistical ranges. Instead, the two chimaera species were scored using the predefined biological thresholds presented in Table 1. This approach avoids treating a sample of *n* = 2 as a statistically stable basis for deriving group-specific reference intervals. The minimum, maximum, median, and observed values underlying the trait-specific classifications are provided in Supplementary Table S1.

For each species, the resilience score was calculated from the seven predefined resilience traits, while the vulnerability score was calculated from the five predefined vulnerability traits. Specifically, R was calculated as the sum of the seven individual resilience-trait scores divided by 7, and V was calculated as the sum of the five individual vulnerability-trait scores divided by 5. Consequently, the denominators of the composite R and V scores were fixed across species. Differences among species in R and V therefore did not result from differences in the number of traits contributing to the respective scores, but from the standardized ordinal scores assigned to the predefined traits.

This fixed-structure approach follows the comparative IRV framework in which multiple biological and fisheries-related characteristics are integrated to provide a standardized species-level assessment [19]. Because the objective was comparative screening rather than estimation of absolute demographic risk, the individual traits were treated within the predefined scoring structure rather than assigned empirically derived differential weights [19,24,25].

### 2.4. Resilience Traits

Resilience was assessed using published key biological traits reflecting species’ capacity to recover from disturbances, including growth coefficient (*K*), asymptotic length (*L_∞_*), length at maturity relative to asymptotic length (*L_m_*/*L_∞_*), gestation period, spawning duration, fecundity, and mean trophic level. These traits are widely used to characterize life-history strategies and recovery potential in fish populations, where faster growth, earlier maturation, higher reproductive output, extended spawning periods, and lower trophic positions are generally associated with higher resilience, while larger body size and slower life-history dynamics are associated with lower resilience [4,32]. These traits were integrated to characterize the relative biological recovery capacity of each species within the assessed assemblage. The resulting R score represents a composite, trait-based measure and should not be interpreted as a direct demographic estimate.

### 2.5. Vulnerability Traits

Vulnerability was assessed as a composite species-level dimension based on geographic distribution, the *L_c_*/*L_∞_* ratio, gear susceptibility, relative population status, and age at maturity. Because these attributes encompass distributional, reproductive, population-status, and fisheries-interaction information, the resulting V score is broader than the fishery-specific susceptibility component used in PSA. Distribution was measured by the number of Mediterranean coastal states reporting each species, with cumulative range area as a proxy; wider ranges received lower scores. The *L_c_*/*L_∞_* ratio indicated capture size relative to asymptotic length, with larger recruitment sizes linked to lower vulnerability. Gear susceptibility was evaluated from species traits and catchability patterns, following proxy-based approaches [25]. Because direct population-size estimates were unavailable for many species, this variable was defined as relative population status and was derived from available evidence on abundance and population trends rather than from geographic range. Geographic distribution was retained as a separate variable. Age at maturity represented reproductive onset, with later maturation increasing vulnerability. These variables were used as relative indicators within the IRV framework and do not constitute a fishery-specific susceptibility score in the conventional PSA sense. Among the variables included in the V component, gear susceptibility represented species interactions with fishing gear, whereas geographic distribution, relative population status, and age at maturity represented distinct distributional, population-level, and reproductive dimensions. Direct measures of spatially and temporally resolved fishing effort or harvest rate were not consistently available across the Mediterranean; therefore, the V score represents a comparative species-level indicator rather than an absolute estimate of risk from a particular fishery.

### 2.6. Determination of the Resilience and Vulnerability Index (IRV) Score

In the resilience–vulnerability (R–V) framework, the position of each species is defined by its resilience (R) and vulnerability (V) scores in a two-dimensional coordinate system. The R–V plot provides the geometric basis of the IRV calculation, with each species represented as a point according to its resilience and vulnerability scores. Two geometric descriptors are used to characterize the position of each species relative to the reference condition: (i) the Euclidean distance from the reference point and (ii) the angular position of the species point relative to the R-axis. These descriptors are used to represent the relative position of species within the R–V space.

The reference point representing the combination of maximum resilience and minimum vulnerability was defined as (3, 1). The R and V scores of each species (x_i_ and y_i_, respectively) were constrained to values between 1 and 3. Thus, the Euclidean distance between the reference point (3, 1) and the position of species (i) was calculated as:

$$d_{i}=\sqrt{{\left(3-x_{i}\right)}^{2}+{\left(y_{i}-1\right)}^{2}}$$

where (d_i_) represents the distance of species (i) from the reference condition.

The angular position (*θ_i_*) was defined relative to the same reference point (3, 1), rather than the absolute coordinate origin. Accordingly, the angle formed by the line connecting the reference point to the species position and the horizontal R-axis was expressed using the translated coordinates:

$$\theta_{i}=\tan^{-1}\left(\frac{y_{i}}{x_{i}}\right)$$

where (*θ_i_*) represents the angular position of species (i) relative to the reference condition. This formulation ensures that both geometric descriptors are defined with respect to the same reference point.

The maximum possible Euclidean distance (d_max_) from the reference point within the R–V space is ($\sqrt{8}$), which corresponds to the distance between the reference point (3, 1) and the opposite corner of the R–V space (1, 3):

$$d_{\max}\;=\;\surd [(3\;-\;1{)}^{2}\;+\;(3\;-\;1{)}^{2}]\;=\;\sqrt{8},$$

The maximum angular component is (π/2) radians. The IRV score, ranging from 0 to 1, was therefore expressed as:

$$\mathrm{IRVi}=\left(1-\frac{d_{i}}{\sqrt{8}}\right)\left(1-\frac{\theta_{i}}{\left(\pi /2\right)}\right)$$

where (IRV_i_) is the integrated resilience–vulnerability score of species (i). Higher IRV values represent positions closer to the reference condition of high resilience and low vulnerability, whereas lower values represent positions farther from this condition [19].

### 2.7. Plotting the R, V and IRV Scores

Two-dimensional R–V scatter plots were generated with resilience (R) scores on the *x*-axis and vulnerability (V) scores on the *y*-axis. The R-axis was displayed in reverse order so that higher resilience values were positioned toward the left side of the plot, whereas higher vulnerability values were positioned toward the upper part of the plot. The IRV score was represented by the size of the points, allowing the integrated position of each species within the R–V space to be visualized simultaneously. Separate plots were generated for sharks, rays, and chimaeras.

Within this representation, positions toward higher R values and lower V values indicate a more favourable resilience–vulnerability position and therefore higher IRV, whereas positions toward lower R values and higher V values indicate a less favourable position and lower IRV. Correlation coefficients (r) and corresponding *p*-values were calculated for the R–V, R–IRV, and V–IRV relationships. Statistical significance was evaluated at α = 0.05.

### 2.8. Comparison of IRV with Other Vulnerability Assessments

The resilience and vulnerability scores obtained from the IRV analysis were compared with the corresponding FishBase evaluations [26]. For descriptive comparison, V scores ranging from 0 to 3 were grouped into low, moderate, high, and very high vulnerability classes, whereas R scores were grouped into very low, low, moderate, and high resilience classes.

The IRV scores were also compared descriptively with the available IUCN Red List categories [27]. IUCN categories were treated as independent conservation-status assessments and were not converted into, or used to define, numerical thresholds for the IRV scores. Accordingly, no direct equivalence was assumed between a particular V-score interval and an IUCN Red List category. Species classified as Data Deficient (DD) were retained as a separate IUCN category because DD indicates insufficient information to assess extinction risk and does not represent a level of extinction risk comparable to Least Concern (LC), Near Threatened (NT), Vulnerable (VU), Endangered (EN), or Critically Endangered (CR). The comparison was therefore intended to examine patterns and differences between the continuous IRV-based vulnerability scores and existing IUCN assessments, rather than to reproduce, replace, or reassign IUCN Red List categories.

## 3. Results

**Figure 1:**
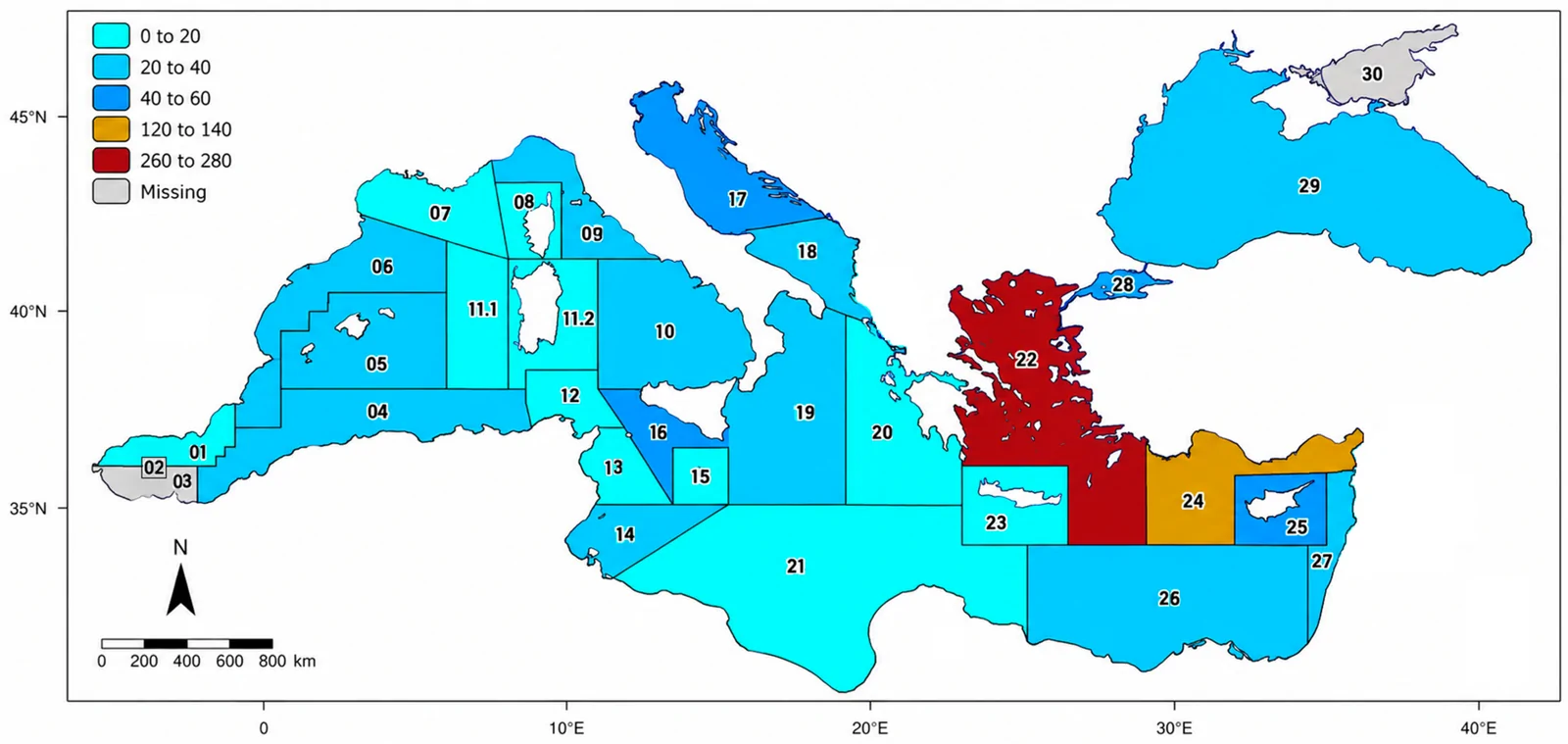
Distribution of the collected raw data records for cartilaginous fishes across the Mediterranean Sea, showing data density within GFCM sub-areas (numbers indicate the respective sub-area codes) according to the colour scale.

The database contained 1634 raw data on 80 cartilaginous species belonging to 47 genera and 30 families in the Mediterranean (Supplementary Table S1. The distribution of the number of collected raw data points on shark, ray, and chimaera species is shown on the GFCM subarea map of the Mediterranean in Figure 1. There was no available data for GFCM subareas 3 and 30, likely due to limited sampling effort and the scarcity of published studies in these regions, whereas the highest amount of raw data was found in GFCM subareas 22 and 24. Species with a documented Mediterranean occurrence and sufficient verified information to assign the predefined R and V trait scores were evaluated within the IRV framework. The dataset, therefore, included species with different regional occurrence patterns, ranging from relatively recurrent species to rare or sporadically recorded taxa. All assessed sharks, rays, and chimaeras were subjected to the same seven-trait resilience and five-trait vulnerability structure, with trait-specific scores assigned according to the predefined criteria in Table 1 [19,26,31].

Across all assessed cartilaginous fish species, the mean resilience (R) and vulnerability (V) scores were 1.82 and 2.31, respectively. Sharks and rays showed similar mean R and V scores, whereas chimaeras had a lower mean R score (1.64) and a lower V score (2.10) than sharks and rays. Mean IRV was highest in sharks (0.17), followed by rays (0.16) and chimaeras (0.16) (Table 2). These descriptive differences reflect the fact that IRV is calculated from the combined geometric position of resilience and vulnerability scores within the R–V space rather than from their arithmetic means.

**Figure 2:**
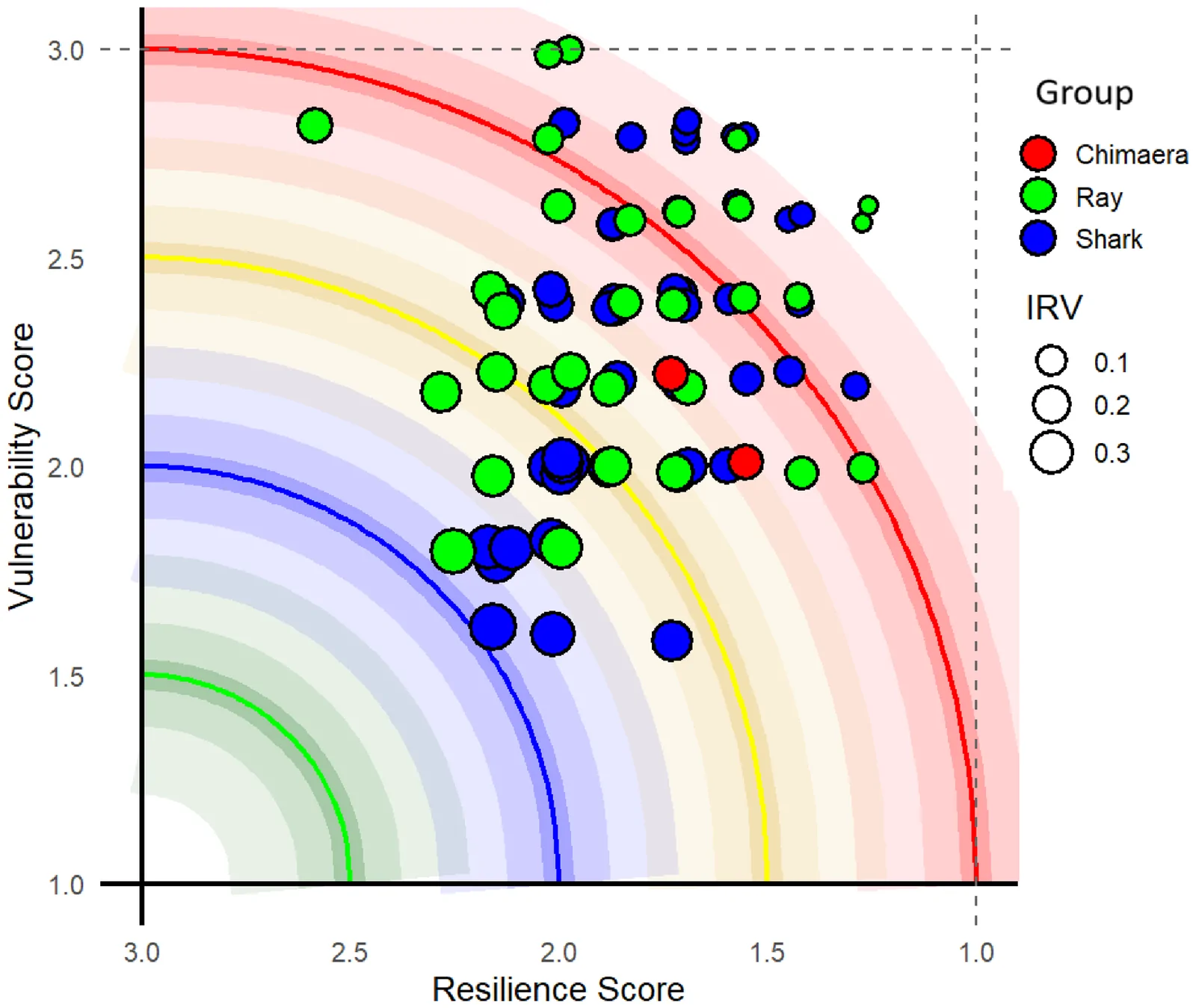
Scatter plot of resilience (R) and vulnerability (V) scores for 80 cartilaginous fish species, including 46 sharks (blue), 32 rays (green), and 2 chimaeras (red). The coloured curves and shaded regions represent a gradient of conservation concern, from green (lower vulnerability and higher resilience, indicating a more favourable status) to red (higher vulnerability and lower resilience, indicating a less favourable status). Point size represents the integrated resilience–vulnerability (IRV) score.

The R–V distribution and corresponding IRV scores of the 80 assessed cartilaginous fish species are presented in Figure 2. The “R”–”V” plot illustrates the distribution of species according to their resilience and vulnerability scores, with point size representing IRV. The concentric curves and shaded regions represent the geometric structure of the IRV space, with positions closer to the high-resilience/low-vulnerability region corresponding to higher IRV values. Resilience scores ranged from 1.29 to 2.57, vulnerability scores ranged from 1.60 to 3.00, and IRV scores ranged from 0.05 to 0.37.

**Figure 3:**
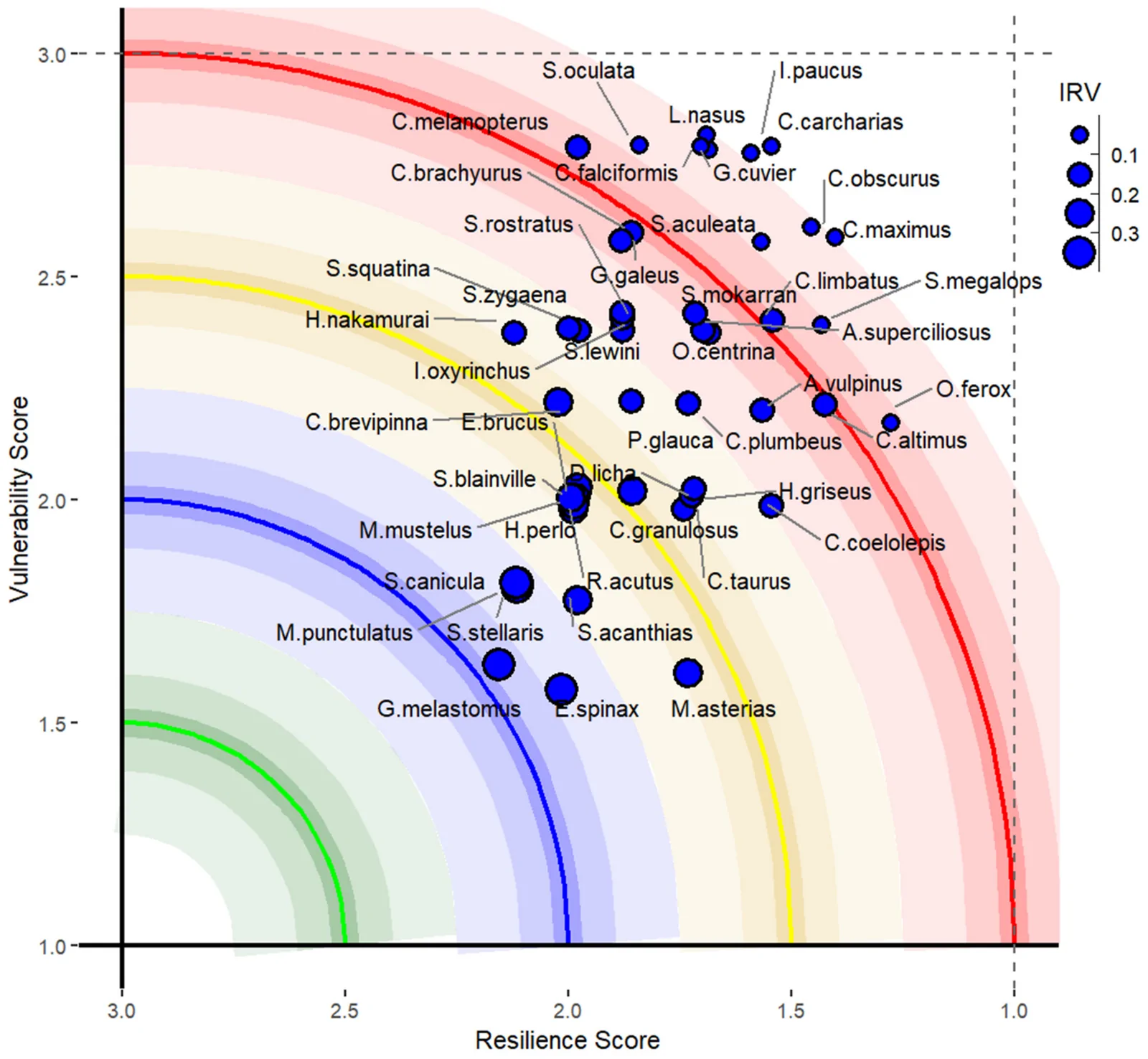
Scatter plot of R-V and IRV scores for shark species. The coloured curves and shaded regions represent a gradient of conservation concern, from green (lower vulnerability and higher resilience, indicating a more favourable status) to red (higher vulnerability and lower resilience, indicating a less favourable status). Point size represents the integrated resilience–vulnerability (IRV) score.

Plotting only the shark species (Figure 3) showed that 10 species had relatively low IRV values (*C. carcharias*, *I. paucus*, *L. nasus*, *G. cuvier*, *C. falciformis*, *O. ferox*, *S. megalops*, *C. maximus*, *C. obscurus*, and *S. oculata*). The least resilient species were *O. ferox*, *C. altimus*, *S. megalops*, and *C. maximus*, whereas the most vulnerable species were *C. melanopterus*, *S. oculata*, *L. nasus*, *G. cuvier*, *C. falciformis*, *C. carcharias*, and *I. paucus*. No shark species was located within the green-shaded region representing comparatively higher resilience and lower vulnerability.

**Figure 4:**
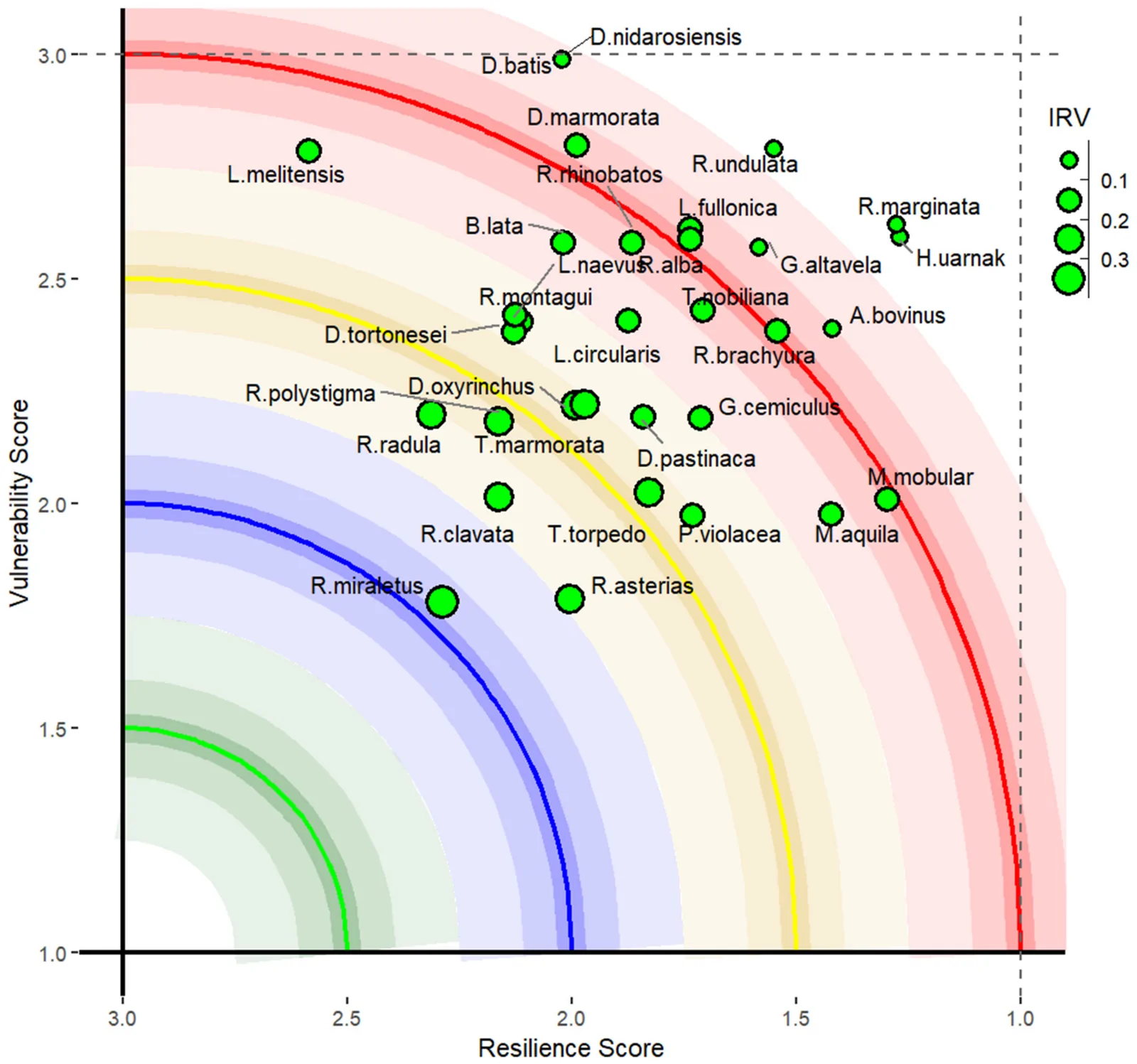
Scatter plot of R-V and IRV scores for ray species. The coloured curves and shaded regions represent a gradient of conservation concern, from green (lower vulnerability and higher resilience, indicating a more favourable status) to red (higher vulnerability and lower resilience, indicating a less favourable status). Point size represents the integrated resilience–vulnerability (IRV) score.

Plotting the ray species (Figure 4) showed that the most vulnerable species were *D. nidarosiensis* and *D. batis*, whereas the least resilient species was *M. mobular*. The species with the lowest IRV scores were *R. undulata*, *R. alba*, *L. fullonica*, *G. altavela*, *H. uarnak*, *R. marginata*, *R. brachyura*, and *A. bovinus*.

**Figure 5:**
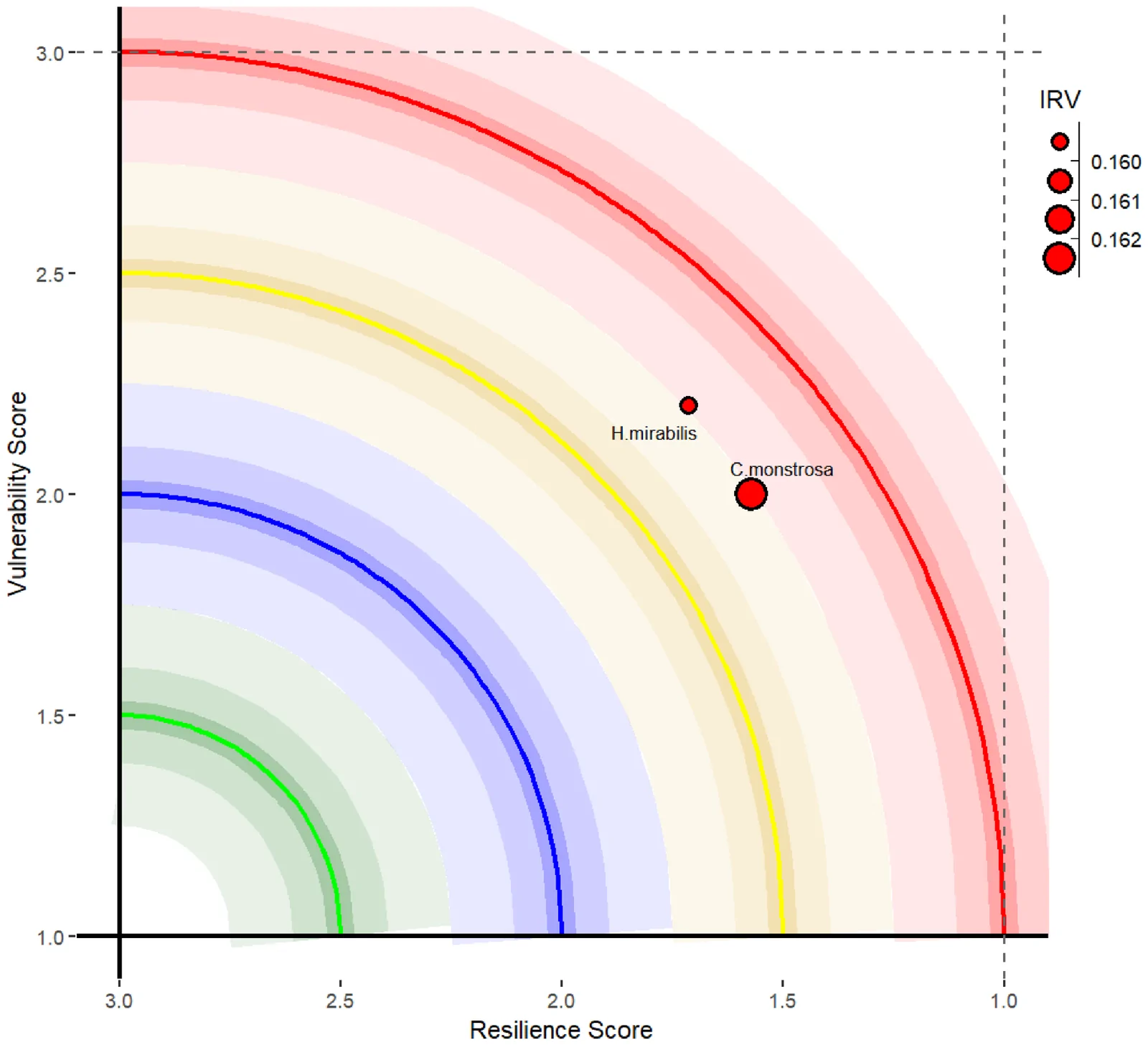
Scatter plot of R-V and IRV scores for chimaera species. The coloured curves and shaded regions represent a gradient of conservation concern, from green (lower vulnerability and higher resilience, indicating a more favourable status) to red (higher vulnerability and lower resilience, indicating a less favourable status). Point size represents the integrated resilience–vulnerability (IRV) score.

Plotting the two chimaera species (Figure 5) showed that *H. mirabilis* had higher resilience and vulnerability scores than *C. monstrosa*, but a lower IRV score.

**Figure 6:**
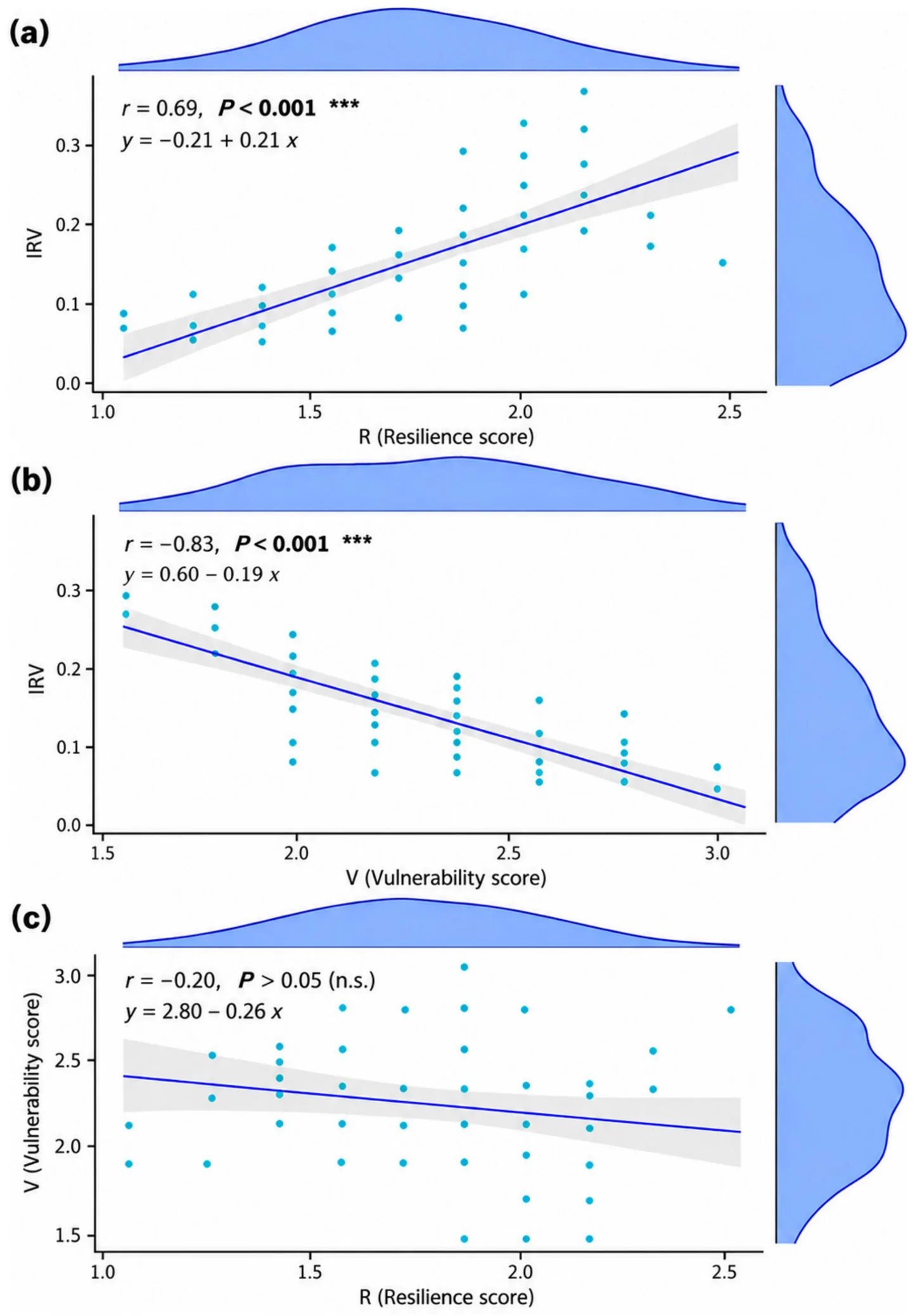
Relationships and distributions among resilience (R), vulnerability (V), and the Index of Resilience and Vulnerability (IRV) for 80 shark, ray, and chimaera species: (**a**) R versus IRV, (**b**) V versus IRV, and (**c**) R versus V. Marginal distributions of the corresponding variables are shown in blue. Points represent individual species, solid lines indicate fitted linear relationships, and shaded areas represent the corresponding 95% confidence intervals. The positive R–IRV and negative V–IRV relationships are inherent to the mathematical construction of IRV, whereas the R–V relationship was not statistically significant (n.s.; *p* > 0.05). *** indicates statistical significance at *p* < 0.001.

The resilience and vulnerability scores showed a weak negative relationship that was not statistically significant (r = −0.20, *p* = 0.065; Figure 6c). In contrast, IRV showed a positive relationship with resilience (r = 0.69, *p* < 0.001; Figure 6a) and a negative relationship with vulnerability (r = −0.83, *p* < 0.001; Figure 6b). Because IRV is mathematically derived from the R and V scores, these latter relationships represent expected properties of the index formulation and should not be interpreted as independent empirical validation of the IRV framework.

The R and V scores of the resilience and vulnerability analysis for 80 species comprising shark, ray, and chimaera species were compared with FishBase global evaluation [26] and the IUCN Mediterranean Sea evaluations [1] for the same species (Table 3). In the FishBase comparison, both assessments showed a predominance of species in the higher vulnerability classes. FishBase classified 33 species as highly vulnerable and 39 as very highly vulnerable, whereas the present IRV analysis classified 38 and 42 species, respectively, in the corresponding broad vulnerability classes (Table 3). For resilience, FishBase classified 34 species as very low resilience and 46 as low resilience, while the present analysis classified 13 and 62 species, respectively, within the corresponding broad resilience classes (Table 4). These comparisons describe the overall frequency distributions of the two assessment approaches and should not be interpreted as evidence of species-level agreement.

Out of the 80 species analysed in our study, 69 were compared with those listed in Dulvy et al. [1] for the current IUCN Mediterranean assessments. Four species, *Carcharhinus brevipinna* [34], *Himantura uarnak* [35], *Sphyrna mokarran* [36], and *S. lewini* [37], were evaluated on a global scale, as Mediterranean-specific assessments were not available. Comparison with the available IUCN Mediterranean assessments [1] showed differences in the distribution of IUCN categories and the corresponding IRV scores (Table 3). Twenty-two species were categorized as LC or NT by the IUCN, whereas the IRV analysis yielded a range of vulnerability scores for these species. In addition, the 13 species categorized as DD by the IUCN also showed a range of IRV scores. These differences should be interpreted cautiously because IUCN Red List categories and IRV scores are based on different assessment frameworks and should not be considered directly equivalent. In particular, the DD category indicates insufficient information to assess extinction risk under the IUCN framework and does not represent a defined level of vulnerability. Therefore, the IRV scores presented here are intended as a complementary, continuous, trait-based assessment and do not constitute a reassignment or replacement of IUCN Red List categories.

The FishBase and present IRV assessments showed broadly similar overall resilience distributions, although the frequencies across resilience classes differed. FishBase classified 34 species as very low resilient and 46 as low resilient, whereas the present analysis classified 10 species as very low resilient and 57 as low resilient. In addition, 12 species were classified as moderately resilient and one as highly resilient in the present analysis, whereas FishBase classified no species as moderately or highly resilient (Table 4).

## 4. Discussion

The IRV analysis enabled the effective aggregation, synthesis, and dissemination of extensive information on life history traits and fisheries impacts across multiple species in the Mediterranean Sea over an extended period. It also helped identify data gaps for key or data-deficient species, guiding the prioritization of biological research to support the management and conservation of sharks, rays, and chimeras. The 80 species covered in this study belonged to 20 families of sharks; 9 families of rays; 1 family of chimaeras. Data remain insufficient for several important species in the Mediterranean, including *Galeus atlanticus*, *Glaucostegus halavi*, *Himantura leoparda*, *Rhincodon typus*, and *Taeniurops grabatus*, as well as for the Mediterranean extinct species *Pristis pectinata* and *P. pristis*. These data-deficient species were not represented in the IRV analysis, as species-level data were available only on a few attributes. A wide range of biological studies is therefore required to fill life-history data gaps for these species.

Although the availability of biological information varies among Mediterranean chondrichthyan species, this did not result in different scoring structures among the species included in the IRV analysis. All assessed sharks, rays, and chimaeras were evaluated using the same predefined seven resilience and five vulnerability traits, following the IRV framework of Mohamed et al. [19] and previous comparative trait-based approaches [26,31]. Each trait was converted into an ordinal score of 1–3 according to the predefined criteria presented in Table 1. The composite R and V scores were calculated using fixed denominators of seven and five, respectively. Therefore, differences in R and V scores were not caused by differences in the number of traits contributing to the scores, but by differences in the standardized trait values assigned to each species. Nevertheless, the uneven availability of underlying biological information remains an important limitation, particularly for poorly studied species, and should be considered when interpreting species-level IRV estimates [19,24,25].

Higher IRV values, considered together with the corresponding R and V scores, indicate a comparatively more favourable combination of higher resilience and lower vulnerability within the IRV framework. Mean IRV scores differed only slightly among the three taxonomic groups, although chimaeras occurred at the comparatively lower end of the IRV range. The R–V plot for sharks showed that 10 species, *C. carcharias*, *I. paucus*, *L. nasus*, *G. cuvier*, *C. falciformis*, *O. ferox*, *S. megalops*, *C. maximus*, *C. obscurus*, and *S. oculata*, had low IRV values and were positioned above the red line. *O. ferox* and *S. oculata* were categorized as CR by the IUCN Red List, while their IRV scores fell within the ranges aligned with EN and CR, respectively. *I. paucus* and *C. maximus* were categorized as DD and EN, respectively, by the IUCN Red List, whereas the IRV scores of both species fell within the range aligned with CR. *C. carcharias* and *L. nasus* were categorized as CR by the IUCN Red List, and their IRV scores also fell within the range aligned with CR. *S. megalops* and *C. obscurus* were categorized as DD by the IUCN Red List, whereas their IRV scores fell within the ranges aligned with EN and CR, respectively. Among rays, *D. nidarosiensis* and *D. batis* had the highest V scores, whereas *M. mobular* had the lowest R score. The species with the lowest IRV scores, *R. undulata*, *R. alba*, *L. fullonica*, *G. altavela*, *H. uarnak*, *R. marginata*, *R. brachyura*, and *A. bovinus*, were positioned above the red line in the R–V plot. *D. batis* and *M. mobular* were categorized as CR and EN, respectively, by the IUCN Red List, whereas their IRV scores fell within the ranges aligned with CR and VU, respectively. *L. fullonica*, *G. altavela*, and *A. bovinus* were all categorized as CR by the IUCN Red List, whereas their IRV scores fell within the ranges aligned with CR, CR, and EN, respectively. *R. alba* and *H. uarnak* were categorized as EN by the IUCN Red List, with the assessment of *H. uarnak* conducted at the global scale, whereas the IRV scores of both species fell within the range aligned with CR. *R. undulata*, *R. marginata*, and *R. brachyura* were categorized as NT, DD, and NT, respectively, by the IUCN Red List, whereas their IRV scores fell within the ranges aligned with CR, CR, and EN, respectively. Differences between IUCN categories and IRV-based classifications indicate that some species require more focused attention and in-depth research; otherwise, they may suffer irreparable consequences.

The scoring framework used in this study offers a way to compare species relative to one another, rather than giving an absolute measure of vulnerability or resilience. It ensures consistent rankings across species but should be understood within the limits of the available data. The scoring method is similar to that used by previous studies [19,24,25].

Although IRV and PSA may incorporate some overlapping biological and fisheries-related traits, they differ in their analytical structure and intended application. PSA combines productivity and susceptibility to assess risk, generally in relation to a defined fishery, whereas IRV separately evaluates biological resilience and broader vulnerability before integrating these dimensions for comparative species-level prioritization. Therefore, the current analysis can be interpreted as an IRV-based Mediterranean-specific assessment rather than a fishery-specific PSA.

In the present analysis, the cut-off criteria set for each trait played a key role in determining the R and V scores. These cut-offs were determined by examining all values across all shark, ray, and chimaera species and dividing them into three ordered categories (1 to 3). For example, the growth coefficient (K) was scored by dividing the highest observed “K” value into three equal parts. As a result, the ranking of each species is relative to the other species in each category—sharks, rays and chimaeras rather than an absolute measure. Owing to the lack of precise abundance estimates, population size was employed as a relative proxy, supported by IUCN Red List data and distribution patterns. Although some species occur in subpopulations that might be considered distinct populations from a general biological perspective, for the purposes of the IUCN criteria, the total number of mature individuals in all Mediterranean areas (i.e., across all subpopulations) is used to determine the species’ population size. It should be noted that for most Mediterranean species, exact population sizes are unknown. Therefore, the best available estimates, based on all accessible data, were used. Furthermore, in the context of this study, species with small areas of occupancy are assumed to likely have small population sizes. Therefore, population size was ranked as abundant population (1), medium-sized population (2), and small population (3). This scoring approach is consistent with methodologies used in previous studies [19,24,25].

The weak and non-significant negative association between resilience and vulnerability (r = −0.20, *p* = 0.065; Figure 6c) suggests that these two dimensions are not necessarily inversely related across Mediterranean chondrichthyans. In other words, species characterized by relatively favourable biological traits are not necessarily those with the lowest vulnerability to fisheries-related pressures. This pattern supports the conceptual distinction between resilience and vulnerability within the IRV framework, in which biological capacity to withstand or recover from disturbance and susceptibility to external pressures represent related but non-equivalent dimensions [19,38,39]. Consequently, a species may exhibit relatively high resilience while still being exposed to substantial fisheries-related vulnerability, depending on its life-history characteristics, fishery interactions, and population status. Conversely, relatively low resilience does not necessarily imply uniformly high vulnerability across all vulnerability-related traits [40].

The relationships observed between IRV and its component scores should be interpreted differently. IRV showed a positive association with resilience (r = 0.69, *p* < 0.001) and a negative association with vulnerability (r = −0.83, *p* < 0.001; Figure 6a,b). These relationships are expected because IRV is mathematically derived from the position of each species within the R–V space. Therefore, the strength and direction of these associations should not be regarded as independent empirical validation of the IRV framework. Rather, Figure 6 illustrates how the calculated IRV values vary geometrically as a function of the underlying resilience and vulnerability scores. The absence of a significant relationship between R and V, in contrast, indicates that the two component dimensions retain a degree of independence within the assessed species assemblage. This distinction is important for interpreting IRV as a composite index: its value lies in integrating two complementary dimensions of biological resilience and vulnerability, rather than in demonstrating an independent relationship between the index and its own constituent variables.

The vulnerability and resilience analysis in this study is broadly comparable with the FishBase [26] assessment, which has been used to characterize intrinsic vulnerability and the potential effects of fishing through trait-based approaches [41]. The frequency distributions of the broad vulnerability classes were generally similar between the two approaches, with both assessments showing a predominance of species in the higher vulnerability classes. Similarly, both assessments indicated that most species occurred within the lower resilience classes, although the number of species assigned to individual classes differed between the two approaches (Table 3 and Table 4). These similarities should be interpreted as similarities in overall frequency distributions rather than as evidence of species-level agreement between the two assessment systems. A formal assessment of agreement would require species-by-species comparison of the corresponding FishBase and IRV scores or categories, which was beyond the scope of the present comparative analysis. Nevertheless, the broadly similar distributional patterns provide useful contextual support for interpreting the relative vulnerability and resilience patterns identified by the IRV framework.

Unlike the trait-based IRV and FishBase assessments, the IUCN Red List evaluates extinction risk using standardized criteria related to population decline, geographic range, population size, and other evidence [1]. The IUCN assessment is extremely useful for designing a conservation plan for a given endangered species but only allows assessment of the vulnerability of a limited number of well-studied species [42]. Further, 16% of 80 shark, ray, and chimaera species in the IUCN Red List in the Mediterranean have been categorised as DD and LC, and 11% of the species have been categorised as NT, which were not directly represented in the IRV classification framework. For example, species that were not evaluated or assessed as DD in the IUCN Red List are assessed in this study CR (*C. brachyurus*, *C. obscurus*, *D. marmorata*, *R. marginata*), EN (*D. tortonesei*, *H. nakamurai*, *C. limbatus*, *C. altimus*, *S. rostratus*) and VU (*S. blainville*) in this study. The vulnerability scores assessed for each shark, ray, and chimaera were also very similar to other vulnerability assessments conducted at a regional scale in the Mediterranean. For example, Saidi et al. [21] assessed the vulnerability of 14 elasmobranchs caught as bycatch in the grouper fishery in the Gulf of Gabès, Tunisia and assessed the vulnerability at a regional scale in the Mediterranean for *C. plumbeus*, *M. mustelus*, *A. bovinus* and *G. cemiculus* as 2.2, 2.0, 2.05, 2.08, respectively; these vulnerability values are 2.2, 2.0, 2.4 and 2.2 for the same species in the present study, respectively.

Taken together, the IRV framework offers a way to compare and rank species based on how resilient or vulnerable they are. Species with relatively lower IRV values may warrant increased attention in future research and monitoring programmes, particularly where independent information on population status and fisheries impacts is limited. However, IRV scores should not be interpreted as direct management priorities or as evidence that species with higher scores require no immediate conservation attention. Rather, the index provides a comparative screening framework that can help identify species for which more detailed population, fisheries, and conservation assessments may be warranted. Management decisions should therefore integrate IRV results with species-specific information on population trends, fishing mortality and effort, habitat use, distribution, exploitation patterns, and formal conservation assessments.

An important consideration in interpreting the present assessment is the heterogeneous occurrence status of chondrichthyan species within the Mediterranean Sea. The regional assemblage includes not only species with relatively well-documented and recurrent occurrence, but also species represented by rare, occasional, or sporadic records [2,6,18]. Such species were retained when their presence in the Mediterranean was supported by reliable published or database records and sufficient information was available for the predefined trait-based assessment. This approach was intended to provide a broad conservation-oriented assessment of the documented Mediterranean chondrichthyan assemblage rather than to define population establishment or residency status. For example, the inclusion of *Galeocerdo cuvier* reflects documented Mediterranean occurrences, including records from Libyan and other Mediterranean waters [43], but should not be interpreted as evidence of a resident or self-sustaining Mediterranean population. Importantly, occurrence frequency itself was not incorporated as a numerical component of the IRV calculation; therefore, the inclusion of rare or sporadic species did not directly increase their vulnerability score. Nevertheless, the limited number of regional observations available for some species represents an important source of uncertainty in interpreting their species-level trait profiles and highlights the need for continued regional monitoring and additional biological and fisheries data [6,18].

The group-level summaries of R, V, and IRV should be interpreted cautiously because the scoring framework is based on ordinal categories that account for the biological ranges of the assessed taxonomic groups. Thus, differences in mean scores among sharks, rays, and chimaeras should not be interpreted as absolute differences in biological resilience or vulnerability. Rather, Table 2 provides a descriptive overview of the scores obtained for the species included in each group. This consideration is particularly important for chimaeras, for which only two species were assessed. Accordingly, the primary interpretation of the IRV framework is based on species-level relative positions within the Mediterranean assemblage, whereas comparisons among taxonomic groups are considered descriptive rather than generalized estimates of group-level resilience or vulnerability.

One major limitation of this study is the lack of direct measures for fishing pressure. Instead, vulnerability was estimated using proxy indicators, which might not fully account for changes in fishing intensity across different areas and times in the Mediterranean. On the other hand, factors tied to resilience, like growth rates and life-history traits, can differ between Mediterranean subregions. Using species-level averages in this study may not accurately capture the variations within specific populations. Hence, the V component is not interpreted as a fishery-specific susceptibility estimate, and the IRV results are used for relative screening and prioritization rather than as a substitute for stock assessment or fishery-specific risk analysis.

## 5. Conclusions

This study highlights the value of the IRV framework as a comprehensive and comparative tool for assessing the life-history traits and fisheries-related risks to sharks, rays, and chimaeras in the Mediterranean Sea. By integrating resilience and vulnerability scores across 80 species, the analysis not only confirmed the high-risk status of many species already recognized as threatened but also identified concerning discrepancies for species currently classified as data deficient or of lower concern by the IUCN. The results indicate the need for targeted biological studies to fill critical data gaps, particularly for understudied or unassessed species. The broad correspondence with FishBase evaluations supports the comparative utility of the IRV results, while differences from some IUCN categories identify species for which additional demographic, fisheries, and population-level investigation may be warranted. The weak relationship between R and V also indicates that favourable biological characteristics do not necessarily protect species from fisheries-related pressures. IRV provides a regional trait-based screening framework that can help identify species warranting further research and monitoring, but it should not be interpreted as a stand-alone basis for management prioritization or as a substitute for fishery-specific risk assessments, stock assessments, or formal conservation-status evaluations.

## Figures and Tables

**Table 2:** The mean R, V and IRV values for cartilaginous fish.

Group	n	R	sd	V	sd	IRV	sd
Shark	46	1.80	0.22	2.27	0.36	0.17	0.08
Ray	32	1.85	0.32	2.38	0.32	0.16	0.08
Chimaera	2	1.64	0.10	2.10	0.14	0.16	0.00
	Mean	1.82	0.26	2.31	0.34	0.17	0.08

**Table 3:** Frequency distributions of FishBase and IUCN assessments and vulnerability classes derived from the present IRV framework for 80 shark, ray, and chimaera species.

**Fishbase Evaluation**		**Present IRV Analyses**		
**Vulnerability State**	**FishBase Scale**	**n**	**V Scale**	**n**
Low	0–25	0	0–0.75	0
Moderate	26–50	8	0.76–1.50	0
High	51–75	33	1.51–2.25	38
Very high	76–100	39	2.26–3.00	42
Total		80		80
IUCN Evaluation *		**Present IRV Analyses**		
**IUCN Scale**		**n**	**V Scale**	**n**
DD		13		0
LC		13	0–0.75	0
NT		9	0.76–1.50	0
VU		14	1.51–2.00	27
EN		12	2.01–2.50	29
CR		19	2.51–3.00	24
Total		80		80

* The 69 species from the list of Dulvy et al. [1], used in our comparisons, were combined with the 11 species not included in that list but evaluated on a global scale in our analyses.

**Table 4:** Comparison of the frequency distributions of FishBase global resilience assessments and the present IRV resilience classes for 80 shark, ray, and chimaera species.

FishBase Evaluation			Present IRV Analyses	
State	FishBase R Scale	n	R Scale	n
Very low	0–25	34	0–1.5	10
Low	26–50	46	1.51–2.00	57
Moderate	51–75	0	2.01–2.50	12
High	76–100	0	2.51–3.00	1
Total		80		80

## Data Availability

The data can be provided upon reasonable request.

## Supplementary Materials

The following supporting information can be downloaded at https://www.mdpi.com/article/10.3390/biology15211931/s1. Supplementary Table S1: Species-level resilience (R), vulnerability (V), and IRV scores, with corresponding FishBase and IUCN assessments. R and V scores and IRV values are those calculated in the present study. FishBase and IUCN fields are provided for comparison and should be completed with the corresponding species-level assessments.
