# Supplementary material for: Evaluating Vulnerability and Resilience of Mediterranean Sharks, Rays and Chimaeras Through Biological and Fishery Traits
Source: Biology (Basel). 2026 Oct 24;15(21):1931. doi: 10.3390/biology15211931 (PMC13762357; doi:10.3390/biology15211931)
Supplement: Supplementary file 1 [file biology-15-01931-s001.zip › biology-4594409-supplementary.pdf]

Supplementary Table S1

Species-level resilience (R), vulnerability (V), and IRV scores, with corresponding FishBase and IUCN assessments. R and V scores and IRV values are those calculated in the present study. FishBase and IUCN fields are provided for comparison and should be completed with the corresponding species-level assessments.

No.	Group	Species	R score	V score	IRV score	FishBase vulnerability	FishBase resilience	IUCN category
1	Shark	Alopias superciliosus	1,714286	2,4	0,130	very high	low	EN
2	Shark	Alopias vulpinus	1,571429	2,2	0,134	very high	low	EN
3	Shark	Carcharhinus plumbeus	1,714286	2,2	0,159	very high	low	EN
4	Shark	Carcharodon carcharias	1,571429	2,8	0,061	very high	very low	CR
5	Shark	Centrophorus granulosus	1,857143	2	0,221	high to very high	very low	CR
6	Shark	Centroscymnus coelolepis	1,571429	2	0,163	high to very high	very low	LC
7	Shark	Cetorhinus maximus	1,428571	2,6	0,066	very high	very low	EN
8	Shark	Dalatias licha	1,714286	2	0,191	high	low	VU
9	Shark	Etmopterus spinax	2	1,6	0,335	high	very low	LC
10	Shark	Galeorhinus galeus	1,857143	2,6	0,120	very high	low	VU
11	Shark	Galeus melastomus	2,142857	1,6	0,373	modarate	low	LC
12	Shark	Heptranchias perlo	2	2	0,250	very high	very low	DD
13	Shark	Hexanchus griseus	1,714286	2	0,191	very high	low	LC
14	Shark	Isurus oxyrinchus	1,857143	2,4	0,151	very high	low	CR
15	Shark	Isurus paucus	1,571429	2,8	0,061	very high	very low	DD
16	Shark	Lamna nasus	1,714286	2,8	0,076	high	very low	VU
17	Shark	Mustelus asterias	1,714286	1,6	0,260	high to very high	very low	VU
18	Shark	Mustelus mustelus	2	2	0,250	very high	very low	VU
19	Shark	Mustelus punctulatus	2,142857	1,8	0,325	modarate to high	low	VU
20	Shark	Oxynotus centrina	1,714286	2,4	0,130	very high	very low	CR
21	Shark	Prionace glauca	1,857143	2,2	0,185	high to very high	low	CR
22	Shark	Scyliorhinus canicula	2,142857	1,8	0,325	high	low	LC
23	Shark	Scyliorhinus stellaris	2,142857	1,8	0,325	very high	low	NT
24	Shark	Somniosus rostratus	1,857143	2,4	0,151	very high	low	DD
25	Shark	Squalus acanthias	2	1,8	0,292	high	very low	VU
26	Shark	Squalus blainville	2	2	0,250	high to very high	very low	DD
27	Shark	Squatina oculata	1,857143	2,8	0,092	very high	low	CR
28	Shark	Squatina squatina	2	2,4	0,173	very high	low	CR
29	Shark	Squatina aculeata	1,571429	2,6	0,084	very high	low	CR
30	Shark	Carcharhinus altimus	1,428571	2,2	0,110	very high	very low	DD
31	Shark	Carcharhinus brachyurus	1,857143	2,6	0,120	very high	very low	DD
32	Shark	Carcharhinus brevipinna	2	2,2	0,210	high	very low	VU
33	Shark	Carcharhinus falciformis	1,714286	2,8	0,076	very high	low	VU
34	Shark	Carcharhinus limbatus	1,571429	2,4	0,108	high	low	DD
35	Shark	Carcharhinus melanopterus	2	2,8	0,107	high	low	VU
36	Shark	Carcharhinus obscurus	1,428571	2,6	0,066	very high	very low	DD
37	Shark	Carcharias taurus	1,714286	2	0,191	high	very low	CR
38	Shark	Echinorhinus brucus	2	2	0,250	very high	very low	EN
39	Shark	Galeocerdo cuvier	1,714286	2,8	0,076	high	low	NT
40	Shark	Hexanchus nakamurai	2,142857	2,4	0,195	very high	very low	DD
41	Shark	Odontaspis ferox	1,285714	2,2	0,088	very high	very low	CR
42	Shark	Rhizoprionodon acutus	2	2	0,250	high	very low	VU
43	Shark	Sphyrna lewini	1,857143	2,4	0,151	very high	low	CR
44	Shark	Sphyrna mokarran	1,714286	2,4	0,130	very high	low	CR
45	Shark	Sphyrna zygaena	2	2,4	0,173	high	low	CR
46	Shark	Squalus megalops	1,428571	2,4	0,087	high	very low	DD
47	Ray	Aetomylaeus bovinus	1,428571	2,4	0,087	very high	very low	CR
48	Ray	Bathytoshia lata	2	2,6	0,139	very high	low	VU
49	Ray	Dasyatis marmorata	2	2,8	0,107	modarate to high	very low	DD
50	Ray	Dasyatis pastinaca	1,857143	2,2	0,185	very high	very low	VU
51	Ray	Dasyatis tortonesei	2,142857	2,4	0,195	very high	very low	DD
52	Ray	Dipturus batis	2	3	0,078	very high	very low	CR
53	Ray	Dipturus nidarosiensis	2	3	0,078	very high	low	EN
54	Ray	Dipturus oxyrinchus	2	2,2	0,210	very high	low	NT
55	Ray	Glaucostegus cemiculus	1,714286	2,2	0,159	high	very low	EN
56	Ray	Gymnura altavela	1,571429	2,6	0,084	modarate	very low	CR
57	Ray	Himantura uarnak	1,285714	2,6	0,050	very high	very low	EN

58	Ray	Leucoraja circularis	1,857143	2,4	0,151	high to very high	low	CR
59	Ray	Leucoraja fullonica	1,714286	2,6	0,102	high to very high	low	CR
60	Ray	Leucoraja naevus	2,142857	2,4	0,195	modarate to high	low	NT
61	Ray	Leucoraja melitensis	2,571429	2,8	0,164	modarate	low	CR
62	Ray	Mobula mobular	1,285714	2	0,109	very high	low	EN
63	Ray	Myliobatis aquila	1,428571	2	0,135	very high	very low	VU
64	Ray	Pteroplatytrygon violacea	1,714286	2	0,191	high	very low	LC
65	Ray	Raja asterias	2	1,8	0,292	low to modarate	low	NT
66	Ray	Raja brachyura	1,571429	2,4	0,108	high	low	NT
67	Ray	Raja clavata	2,142857	2	0,279	high	low	NT
68	Ray	Raja miraletus	2,285714	1,8	0,357	high	low	LC
69	Ray	Raja montagui	2,142857	2,4	0,195	high	low	LC
70	Ray	Raja polystigma	2,142857	2,2	0,235	high	low	LC
71	Ray	Raja radula	2,285714	2,2	0,259	modarate to high	low	EN
72	Ray	Raja undulata	1,571429	2,8	0,061	high to very high	low	NT
73	Ray	Rhinobatos rhinobatos	1,857143	2,6	0,120	high to very high	low	EN
74	Ray	Rhinoptera marginata	1,285714	2,6	0,050	very high	very low	DD
75	Ray	Rostroraja alba	1,714286	2,6	0,102	very high	low	EN
76	Ray	Tetronarce nobiliana	1,714286	2,4	0,130	very high	low	LC
77	Ray	Torpedo marmorata	2	2,2	0,210	high	low	LC
78	Ray	Torpedo torpedo	1,857143	2	0,221	high	low	LC
79	Chimaera	Chimaera monstrosa	1,571	2	0,162	high to very high	low	NT
80	Chimaera	Hydrolagus mirabilis	1,714	2,2	0,159	modarate	low	LC
